# Evaluation of Thyroid Function Tests in Patients With COVID-19

**DOI:** 10.7759/cureus.40628

**Published:** 2023-06-19

**Authors:** Cemalettin Durgun, Mehmet Durgun

**Affiliations:** 1 General Surgery, Memorial Dicle Hospital, Diyarbakır, TUR; 2 Pulmonology, Dağkapı State Hospital, Diyarbakır, TUR

**Keywords:** covid-19, free t3, free t4, mortality, thyroid-stimulating hormone (tsh), thyroid function

## Abstract

Background

SARS-CoV-2 infects cells via angiotensin-converting enzyme 2 (ACE2). ACE2 levels are high in the thyroid gland. Although the thyroid gland can be directly infected in COVID-19 patients, the hypothalamic-pituitary-thyroid axis is also affected. Therefore, changes in thyroid function occur in COVID-19 patients. This study aimed to examine the effect of thyroid function tests on the prognosis of COVID-19.

Methodology

A total of 146 patients who were diagnosed with COVID-19 and treated in the intensive care unit between August and November 2021 and who had no previous history of thyroid disease were included in the study. Demographic information, laboratory tests, and thyroid hormone levels during hospitalization and discharge patterns were evaluated. The patients were divided into two groups: group I included those who were discharged after recovery, and group II included those who did not respond to treatment and died.

Results

When the fT3 and fT4 levels of the patients were compared, the hormone levels decreased as the clinical severity of the disease increased. The amount of decrease in hormone levels was mostly seen in group II. In the recovered patient group, the amount of hormone decreased was less. The difference between fT3 and ft4 values between the groups was found to be statistically significant (*P *= 0.015 and *P *= 0.004). In addition, the difference between the groups' C-reactive protein (CRP), D-dimer, and ferritin values was statistically significant (*P *= 0.036, *P *= 0.022, and *P *< 0.000, respectively). There was no statistically significant difference between the groups in terms of demographic characteristics (*P *> 0.05).

Conclusions

Thyroid hormone changes were found to be an important prognostic parameter affecting disease severity and mortality in COVID-19 patients and can be used to predict mortality.

## Introduction

COVID-19) caused by SARS-CoV-2, which progresses with severe acute respiratory syndrome, first appeared in China and soon turned into a pandemic and spread all over the world [1]. Although the main target organs of the virus are considered to be the respiratory system and the immune system, it affects all systems, including the endocrine system [2]. SARS-CoV-2 enters the body through the upper respiratory tract mucosa and incubates for 5-14 days [3]. Usually, symptoms such as fever of varying degrees, respiratory tract symptoms, myalgia, cough, and malaise occur. However, some patients experience serious health problems such as respiratory failure, acute respiratory distress (ARDS), sepsis, and myocarditis, more frequently in people with comorbid diseases such as chronic kidney failure, diabetes, and heart diseases [4,5]. SARS-CoV-2 affects all systems as well as the thyroid gland. SARS-CoV-2 enters cells via angiotensin-converting enzyme 2 (ACE2). ACE2 is a functional receptor located on the cell wall. This enzyme is concentrated in the heart, kidneys, lungs, and thyroid gland. Although the thyroid gland is directly affected in patients infected with SARS-CoV-2, the hypothalamic-pituitary-thyroidal pathway is also impaired.

Many prognostic factors play a role in the severity of the disease. Meta-analyses report that the serum ferritin level is an independent risk factor in determining disease severity in patients with COVID-19. C-reactive protein (CRP), interleukin-6 (IL-6), D-dimer, and lymphocyte count are reported as other prognostic risk factors [6].

This study was conducted to investigate whether thyroid function tests play a role in disease severity as a prognostic factor.

## Materials and methods

This retrospective study was conducted with the permission of the Ministry of Health of the Republic of Turkey. It was approved by the Ethics Committee of Memorial Şişli Hospital with the decision numbered 24.12.2021/008. The research was conducted per the Declaration of Helsinki.

Between August and November 2021, 146 patients with a laboratory and clinical diagnosis of COVID-19 who were hospitalized and treated in the intensive care unit, had euthyroid thyroid function tests, and no history of thyroid gland disease were included in the study.

Demographic characteristics and changes in thyroid hormones, D-dimer, CRP, and ferritin levels during hospitalization were evaluated. Patients who were positive for SARS-CoV-2 in the quantitative reverse transcriptase polymerase chain reaction were considered COVID-19 positive.

Patients were divided into two groups: group I included patients treated in the intensive care unit and discharged after recovery, and group II included patients who did not respond to treatment and died.

The treatment algorithms recommended by the Ministry of Health and the Scientific Committee were applied to the patients. After the diagnosis of COVID-19, all patients were followed up by examining their biochemical parameters and thyroid function tests. Pulmonary involvement was evaluated and followed up by chest computed tomography (CT). Patients with respiratory rate ≥30 breaths/minute, resting oxygen (O_2_) saturation ≤85%, and arterial partial oxygen pressure (PaO_2_)/oxygen concentration (FiO_2_) ≤ 250 mmHg were followed up in the intensive care unit. In addition to symptomatic treatment, high-flow continuous oxygen therapy was given. Despite the treatment, patients whose O_2_ saturation fell below 75, who developed organ failure, and whose lung involvement progressed on CT were intubated. Thyroid-stimulating hormone (TSH), free triiodothyronine (fT3), free thyroxine (fT4), D-dimer, CRP, and ferritin levels of the patients were followed up at 48-hour intervals.

Statistics

Data were analyzed in the Statistical Package for Social Sciences version 21.0 (SPSS Inc., Chicago, IL, USA). The Mann-Whitney U test between continuous variables was used for comparison between the two groups. The chi-square test was used to evaluate categorical variables. It was considered statistically significant when the *P*-value < 0.05.

## Results

Within the scope of the research, it was examined whether there was a difference between thyroid functions, D-dimer, ferritin, and CRP levels according to the discharge type from the hospital. The research data pool consists of 146 patients, of which 45.9% were female and 54.1% were male. Descriptive statistics regarding the values obtained from the patients are given in Table 1.

**Table 1:** Death/recovery discharge distribution table by gender. Pearson chi-square = 3.817;  *P *= 0.051.

			Status		Total
			Recovery	Death	
Gender	Female	n	38	29	67
		%	56.7	43.3	100
	Male	n	32	47	79
		%	40.5	59.5	100
Total		n	70	76	146
		%	47.9	52.1	100

The distribution of recovery or death status according to gender did not show a statistically significant change.

As a result of the study, it was observed that there was a significant difference between the groups in terms of fT3 and fT4 values. A greater decrease in fT3 (*P* = 0.015) and fT4 (*P* = 0.004) values was observed in the deceased group. There was no significant difference in TSH values between the groups according to the discharge status of the patients. The difference between the groups was not statistically significant (*P *= 0.883).

There was a statistically significant difference between the groups in terms of D-dimer values (*P *= 0.022). It was determined that the group with the highest D-dimer value was the death group. The group discharged with recovery had the lowest D-dimer value.

There was a statistically significant difference between the groups in terms of CRP values (*P *= 0.036). It was observed that the group with the highest CRP value was the death group.

There was a statistically significant difference between the groups in terms of ferritin values (*P *< 0.001). The ferritin value of the deceased group was higher than the ferritin value of the recovered group.

No statistically significant difference was found between the groups in terms of age and number of days of hospitalization. The results of the analysis are shown in Table 2.

**Table 2:** Statistical evaluation table of parameters for death-recovery status. TSH, thyroid-stimulating hormone, fT3, free triiodothyronine; fT4, free thyroxine

Parameter	Discharge status	n	Mean	Mean rank	Median	Minimum	Maximum	Mann-Whitney U-test	P
Age (years)	Recovery	70	59.87	72.76	63.000	22	91	2608.000	0.839
	Death	76	60.32	74.18	59.500	23	95		
Number of hospitalization days	Recovery	70	8.77	67.89	7.000	1	31	2267.500	0.123
	Death	76	10.59	78.66	8.500	0	31		
CRP	Recovery	70	73.8757	65.87	44.850	0.20	300,.0	2126.000	0.036
	Death	76	99.1000	80.53	87.950	0.20	329.70		
D-dimer	Recovery	70	1.7897	64.64	0.850	0.15	10.00	2045.500	0.022
	Death	76	3.6432	80.59	1.700	0.15	10.00		
Ferritin	Recovery	70	639.9857	59.89	429.000	26.00	2000.00	1707.500	0.000
	Death	76	1045.7763	86.03	883.000	41.00	2000.00		
fT3	Recovery	70	1.7967	81.76	1.620	1.50	3.46	2082.000	0.015
	Death	76	1.6421	65.89	1.500	1.50	2.63		
fT4	Recovery	70	1.0597	84.01	1.040	0.69	1.56	1924.000	0.004
	Death	76	0.9420	63.82	0.970	0.40	1.46		
TSH	Recovery	70	0.8916	74.04	0.395	0.00	10.83	2622.500	0.883
	Death	76	1.3583	73.01	0.380	0.01	44.25		

## Discussion

ACE2 plays a critical role in the entry of the SARS-CoV-2 virus into the host [7]. The virus settles in the body by binding to the ACE2 receptors on the host cell membrane. The thyroid, pancreas, intestine, testis, ovaries, adrenal glands, and pituitary are endocrine organs that express ACE2 [8]. These data, in line with our study, explain the development of hypothyroidism in patients with COVID-19. SARS-CoV-2 invades target organs, deforms cells, and prevents the tissue from performing its normal function.

Abnormal thyroid hormone levels can be observed in patients due to some serious diseases other than thyroid gland diseases, and this condition is called nonthyroidal illness syndrome (NTIS) or euthyroid sick syndrome (ESS) [9]. The most common causes of NTIS are serious infections, liver and kidney failure, complications of diabetes, malignant tumor, and malnutrition. The most typical changes are decreased T3 level, low or normal T4 level, and normal or slightly decreased TSH level. These changes vary according to the severity of the underlying disease [10]. As severe COVID-19 cases in need of intensive care were evaluated in our study, T3, T4, and TSH levels were low in most patients. This shows that the SARS-CoV-2 virus causes multiorgan failure in severe disease form. A previous study revealed that the thyroid follicle and parafollicular cells of patients with SARS were affected in correlation with the severity of the disease [11]. Wei et al. showed that the number of TSH-positive cells and staining intensity were reduced in the pituitary gland of SARS patients [12]. In their study, Chen et al. showed that TSH and T3 levels in patients with COVID-19 were low and inversely proportional to the severity of the disease [13]. The fact that serum TSH levels are lower in patients with COVID-19 compared to other pneumonia patients indicates that the SARS-CoV-2 virus is more effective on the pituitary. In our study, in accordance with the literature, TSH, fT3, and fT4 levels were found to be low in patients with COVID-19 who needed intensive care.

In their retrospective study, Lang et al. emphasized that a low FT3 level at the time of admission to the hospital is a risk for mortality in patients with COVID-19 [14]. Our study showed that the decrease in thyroid function tests in previously euthyroid patients was correlated with the course of the disease.

Besides the direct effect of SARS-CoV-2, it also affects thyroid function secondary to the cytokine storm caused by the infection, resulting in thyroid hormone dysfunction during COVID-19 [15,16]. In previous studies, it has been shown that COVID-19 directly affects the pituitary cells that secrete TSH.

In addition, the pituitary gland may be affected by systemic action mediated by the activation of proinflammatory cytokines through chronic hypoxic stress or in patients using glucocorticoids. They also showed that SARS-CoV-2 infection may affect the function of the thyroid gland secondary to thyroid damage and atypical thyroiditis or cytokine storm [16,17].

Serum ferritin is both an indicator of iron stores in the body and an inflammatory marker. Hyperferritinemia caused by excessive inflammation correlates with the severity of the infection. It is a biomarker by which we can evaluate the response of the infection to treatment. High ferritin levels are associated with mortality [18,19]. High serum ferritin level, which is a feature of hemophagocytic lymphohistiocytosis resulting from a viral infection, is closely related to the poor prognosis of patients with COVID-19, and high ferritin level is correlated with the increase in lung lesions [20]. Cheng et al. showed in their meta-analysis that the serum ferritin level of patients with COVID-19 is associated with disease severity, mortality, and response to treatment [21]. Huang et al. stated in their study that high serum CRP, Procalcitonin, D-dimer, and ferritin levels were associated with a poor prognosis of COVID-19 [22]. When the data we obtained in our study are compared with the literature, it is seen that they are compatible. In our study, it is seen that thyroid function tests show a negative correlation with the serum ferritin level, which is stated as an independent risk factor in determining the severity of the disease, and the decrease in fT4 and fT3 levels becomes more pronounced as the disease severity increases.

This study has certain limitations. First, it is a retrospective study. Second, the scope of the study is limited to patients treated in the intensive care unit, which inherently restricts the sample size and may limit the generalizability of the findings.

## Conclusions

This study showed that most critically ill patients with COVID-19 infection had abnormal thyroid function fests. fT3 and fT4 concentrations were significantly lower in patients who died due to COVID-19 than in those who recovered. Many prognostic factors play a role in disease severity. Thyroid function tests seem to be an important parameter that can be used together with other prognostic factors to predict disease severity and mortality.

In patients hospitalized for COVID-19, we recommend that clinicians closely monitor fT3 and fT4 as markers of potential progression to critical illness.

## References

[1] Guan WJ, Ni ZY, Hu Y (2020). Clinical characteristics of coronavirus disease 2019 in China. N Engl J Med.

[2] Pathak SK, Pandey S, Pandey A, Salunke AA, Thivari P, Ratna HV, Chawla J (2020). Focus on uncommon symptoms of COVID-19: potential reason for spread of infection. Diabetes Metab Syndr.

[3] Pal R, Banerjee M (2020). COVID-19 and the endocrine system: exploring the unexplored. J Endocrinol Invest.

[4] Zheng Z, Peng F, Xu B (2020). Risk factors of critical &amp; mortal COVID-19 cases: a systematic literature review and meta-analysis. J Infect.

[5] Zhang JJ, Dong X, Cao YY (2020). Clinical characteristics of 140 patients infected with SARS-CoV-2 in Wuhan, China. Allergy.

[6] Henry BM, de Oliveira MH, Benoit S, Plebani M, Lippi G (2020). Hematologic, biochemical and immune biomarker abnormalities associated with severe illness and mortality in coronavirus disease 2019 (COVID-19): a meta-analysis. Clin Chem Lab Med.

[7] Liu M, Wang T, Zhou Y, Zhao Y, Zhang Y, Li J (2020). Potential role of ACE2 in coronavirus disease 2019 (COVID-19) prevention and management. J Transl Int Med.

[8] Liu F, Long X, Zhang B, Zhang W, Chen X, Zhang Z (2020). ACE2 expression in pancreas may cause pancreatic damage after SARS-CoV-2 ınfection. Clin Gastroenterol Hepatol.

[9] Fliers E, Bianco AC, Langouche L, Boelen A (2015). Thyroid function in critically ill patients. Lancet Diabetes Endocrinol.

[10] Van den Berghe G (2014). Non-thyroidal illness in the ICU: a syndrome with different faces. Thyroid.

[11] Wei L, Sun S, Xu CH (2007). Pathology of the thyroid in severe acute respiratory syndrome. Hum Pathol.

[12] Wei L, Sun S, Zhang J (2010). Endocrine cells of the adenohypophysis in severe acute respiratory syndrome (SARS). Biochem Cell Biol.

[13] Chen M, Zhou W, Xu W (2021). Thyroid function analysis in 50 patients with COVID- 19: a retrospective study. Thyroid.

[14] Lang S, Liu Y, Qu X (2021). Association between thyroid function and prognosis of COVID- 19: a retrospective observational study. Endocr Res.

[15] Muller I, Cannavaro D, Dazzi D (2020). SARS-CoV-2-related atypical thyroiditis. Lancet Diabetes Endocrinol.

[16] Gorini F, Bianchi F, Iervasi G (2020). COVID-19 and thyroid: Progress and prospects. Int J Environ Res Public Health.

[17] Lania A, Sandri MT, Cellini M, Mirani M, Lavezzi E, Mazziotti G (2020). Thyrotoxicosis in patients with COVID-19: the THYRCOV study. Eur J Endocrinol.

[18] Kernan KF, Carcillo JA (2017). Hyperferritinemia and inflammation. Int Immunol.

[19] Carcillo JA, Sward K, Halstead ES (2017). A systemic ınflammation mortality risk assessment contingency table for severe sepsis. Pediatr Crit Care Med.

[20] Mehta P, McAuley DF, Brown M, Sanchez E, Tattersall RS, Manson JJ (2020). COVID-19: consider cytokine storm syndromes and immunosuppression. Lancet.

[21] Cheng L, Li H, Li L, Liu C, Yan S, Chen H, Li Y (2020). Ferritin in the coronavirus disease 2019 (COVID-19): a systematic review and meta-analysis. J Clin Lab Anal.

[22] Huang I, Pranata R, Lim MA, Oehadian A, Alisjahbana B (2020). C-reactive protein, procalcitonin, D-dimer, and ferritin in severe coronavirus disease-2019: a meta-analysis. Ther Adv Respir Dis.

